# Lung cancer presenting as heel pain: A case report

**DOI:** 10.3892/ol.2014.2164

**Published:** 2014-05-22

**Authors:** HAO DAI, MINFEI QIANG, YANXI CHEN, WEITAO ZHAI, KUN ZHANG

**Affiliations:** 1 Department of Orthopedics, Guanghua Integrative Medicine Hospital, Shanghai 200052, P.R. China; 2 Department of Orthopedics and Traumatology, East Hospital, Tongji University School of Medicine, Shanghai 200120, P.R. China

**Keywords:** heel pain, lung cancer, biopsy, bone metastasis

## Abstract

Bone metastasis as the first symptom of lung cancer is common, particularly in the axial skeleton. The calcaneus is an unusual site of metastatic involvement. Chronic plantar heel pain (CPHP) is one of the most common complaints of the foot requiring medical treatment. The most typical symptom of CPHP is pain under the medial heel during weight-bearing, and this symptom is therefore generally initially diagnosed as CPHP by clinicians. The current case study reports a female patient never-smoker with non-small cell lung cancer accompanied by calcaneal metastasis presenting as heel pain. The patient was initially diagnosed with CPHP without any imaging examinations. As there was no relief from the heel pain six months later, a foot X-ray was performed, which revealed a lesion of the calcaneus. The analysis of a biopsy obtained from the lesion resulted in a diagnosis of adenocarcinoma. The present case indicates that patients suspected to have CPHP should be conventionally examined with radiography of the foot during the initial diagnosis. Similarly, if a patient with lung cancer has symptoms such as CPHP, distant metastasis should be accounted for; despite their rarity, clinicians should maintain a high level of suspicion, since accurate diagnosis and timely treatment is important in management and outcome.

## Introduction

Bone metastasis can occur as the first symptom of lung cancer, and >80% of metastases are in the axial skeleton, including the spine, ribs and pelvis (1). A retrospective review of 259 patients with non-small cell lung cancer (NSCLC) reported that 30.4% of patients were found to have bone metastases during their clinical course and that 50% of patients with bone metastases suffered from skeletal-related events (2). The calcaneus is an unusual site of metastatic involvement. Metastases to the bone develop in 30% of all patients with cancer, with only 0.007–0.3% exhibiting acrometastases (3). Heel pain, or calcaneodynia, is a frequent symptom in patients with foot and ankle disorders. Plantar fasciitis (PF) is the most common cause of heel pain and one of the most common conditions for which patients with foot pain seek medical treatment (4). A previous study reported that 10% of the population suffer from this condition at any given time (5). The current study reports a rare case of a patient with lung cancer that metastasized to the calcaneus. The treatment had been delayed for six months with a misdiagnosis of chronic plantar heel pain (CPHP). Patient provided written informed consent.

## Case report

In July 2012, a 58-year-old female was admitted to the Department of Orthopedics of the Guanghua Integrative Medicine Hospital, Changning District (Shanghai, China) due to left plantar heel pain without any inducing factors. The symptom was aggravated for six months when walking and standing. The patient had been diagnosed with CPHP in a local hospital without any auxiliary examination six months prior to admittance. There had been no obvious relief of the symptom following anti-inflammatory and analgesic treatment. The patient was admitted to the Guanghua Integrative Medicine Hospital, Changning District after the heel pain became increasingly severe for several days, particularly during weight-bearing. The patient had no fever, radiating pain to the left lower limb, night pain or numbness of local skin. Examination demonstrated that the skin on each side of the left calcaneus was slightly deep in color and the soft tissue was mildly swollen. Tenderness was identified at the end of the plantar fascia below the heel. There was no paresthesia of the surrounding skin, and the motion of the foot and ankle was normal. The patient was healthy with a normal diet prior to the emergence of the symptom. There was no progressive weight loss. The patient had never smoked and had no cough, expectoration or hemoptysis and denied having contracted any contagious diseases.

**Figure 1:**
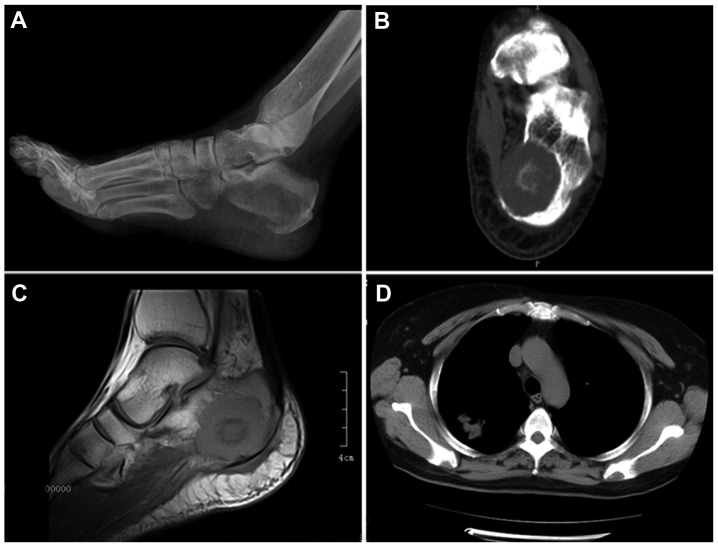
Medical imaging examinations including (A) an X-ray of the left calcaneus; (B) a computed tomography (CT) scan of left calcaneus; (C) a sagittal MR T1-weighted image of the left calcaneus; and (D) chest CT scans of the patient. MR, magnetic resonance.

**Figure 2:**
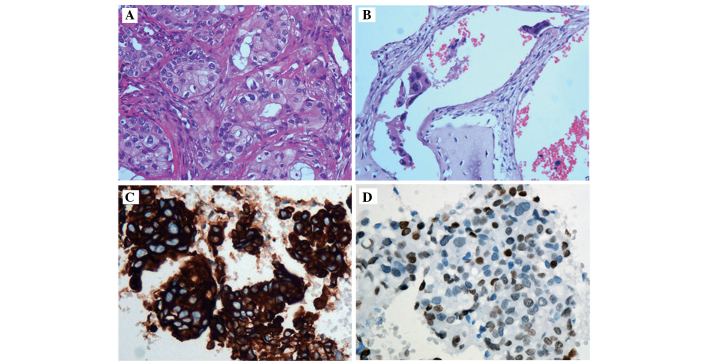
Histological specimens obtained from a biopsy of the calcaneal lesion. (A) The cancer cells tended to nest during growth and had obvious atypia with big nuclei, visible nucleoli and rich pink-stained cytoplasm. Adenoid lacunae were observed among the cancer cells. There were dense fibrous tissues between the cancer cell nests (hematoxylin-eosin stain; original magnification, ×200). (B) A cancer embolus could be observed in the blood vessel (hematoxylin-eosin stain; original magnification, ×200). (C) The cancer cells were positive for CK7 (envision method; original magnification, ×200). (D) The cancer cells were positive for thyroid transcription factor 1 (TTF1; envision method; original magnification, ×200). CK7, cytokeratin 7.

Foot and ankle radiographs showed spurs in the left heel and an osteolytic lesion of the calcaneus (Fig. 1A). A computed tomography (CT) scan revealed an occupied lesion of the calcaneus with bone destruction, which was considered to be a malignant tumor (Fig. 1B). Magnetic resonance imaging indicated the same result (Fig. 1C). The chest CT scan presented an occupied lesion in the posterior segment of the right upper lung lobe, considered as a peripheral lung cancer (Fig. 1D). A bone scan showed abnormalities in the left calcaneus. The results of routine blood analyses of the erythrocyte sedimentation rate, C-reactive protein and alkaline phosphatase levels, and liver and kidney functions were normal. An open biopsy of the calcaneus lesion performed under general anesthesia showed a poorly-differentiated adenocarcinoma. The tumor cells were positive for cytokeratin (CK) 7, thyroid transcription factor 1, 34βE12, CK8, p53 and cluster of differentiation 34 in vessels (Fig. 2). The patient then received left calcaneus lesion curettage and replacement with bone cement. The surgery was successful and the wound healed primarily. Subsequently, the patient received palliative care, but succumbed to NSCLC six months after the accurate diagnosis.

## Discussion

CPHP is one of the most frequent complaints of the foot and is responsible for 15% of all adult foot complaints that require medical treatment. CPHP is prevalent in athletic and non-athletic populations (6). The etiology of CPHP is poorly understood and is possibly multifactorial, including heel spurs, pes planus and systemic disorders, but particularly plantar fasciitis (7). The pain is generally observed in patients that are >40 years of age. The typical complaint shared by the majority of individuals is pain under the medial heel during weight-bearing, particularly in the morning and at the beginning of weight-bearing activities such as walking (8). In the present case, the first symptom was a gradual onset of pain in the medial heel that was usually worse with the first step in the morning or following a period of inactivity. Upon first presentation, the patient did not receive any image examination for the foot; this is a common occurrence, therefore metastasis to the foot tends to be misdiagnosed by clinicians as CPHP.

Acrometastases (metastases to the hand or foot) occur rarely, however, bones are typical sites of disease metastasis that occurs in ≤30% of patients with malignancy (3). In Western countries, it has been reported that the incidence of bone metastases in patients with lung cancer is 30–40%, and >80% of them are in the axial skeleton, including the spine, ribs and pelvis (1). The median survival time of patients with such metastases is seven months (9). McGarry (10) reported that a 45-year-old male with a 70-pack-year history of smoking was affected with primary bronchogenic carcinoma metastasis to the calcaneus. The patient succumbed 21 months after the diagnosis. There have been no other cases reported with regard to lung cancer presenting as a calcaneal metastasis. However, the patient of the current study had no history of smoking and was healthy prior to presentation. The treatment had been delayed for six months with a misdiagnosis of CPHP. When the metastatic tumor of the calcaneus was found, it had become a poorly-differentiated adenocarcinoma with a higher degree of malignancy. The cancer cells grew invasively in the bone and cartilage and a tumor thrombus could be observed in the vessel. The prognosis of lung cancer is closely associated with early diagnosis and intervention. Without an early diagnosis in the patients with a disease procession similar to the patient of the present study, the risks of mortality will increase. Therefore, we suggest that patients with suspected CPHP should be conventionally examined with a foot and ankle X-ray during their first diagnosis. A high degree of attention must be paid if there is an osteolytic lesion.

In conclusion, as reported in the present case, metastatic lung cancer can initially be misdiagnosed as CPHP, which may lead to a poor prognosis. Although the calcaneus is a rare metastatic site of disease, it may cause the only and initial symptoms. In addition, if a patient with lung cancer has symptoms such as CPHP, a distant metastasis must be accounted for. Clinicians should keep an open mind when a patient presents with unrelated symptoms. Despite their rarity, clinicians should maintain a high level of suspicion, since accurate diagnosis and timely treatment is important in the management and outcome of the condition.
